# Epidemiological and Clinical Aspects of Cutaneous and Mucosal Leishmaniases in Portugal: Retrospective Analysis of Cases Diagnosed in Public Hospitals and Reported in the Literature between 2010 and 2020

**DOI:** 10.3390/microorganisms12040819

**Published:** 2024-04-18

**Authors:** Rafael Rocha, Cláudia Conceição, Luzia Gonçalves, Ana Cláudia Carvalho, André Maia, André Martins, António Carujo, António Maio, Catarina Forra, Catarina Melita, Daniela Couto, Diana Fernandes, Dulce Pereira, Ema Leal, Helena Sarmento, Inês Sousa, Jean-Pierre Gonçalves, Joana Marinho, Joana Vasconcelos, João Cunha, João Rodrigues, José Miguel Silva, Lídia Caley, Luís Malheiro, Luís Santos, Margarida Garcia, Maria Cunha, Maria Lima, Maria Margarida Andrade, Marta Marques, Miguel Alpalhão, Mónica Silva, Rita Ferraz, Rui Soares, Salomão Fernandes, Samuel Llobet, Sofia Cruz, Teresa Guimarães, Tiago Branco, Tomás Robalo-Nunes, Vasco Almeida, Carla Maia

**Affiliations:** 1 Instituto de Higiene e Medicina Tropical (IHMT), Universidade Nova de Lisboa (UNL), Rua da Junqueira N°100, 1349-008 Lisboa, Portugal; rafael.amorim.rocha@gmail.com (R.R.); claudiaconceicao@ihmt.unl.pt (C.C.);; 2 Global Health and Tropical Medicine (GHTM), Associate Laboratory in Translation and Innovation Towards Global Health (LA-REAL), Instituto de Higiene e Medicina Tropical (IHMT), Universidade Nova de Lisboa (UNL), Rua da Junqueira N°100, 1349-008 Lisboa, Portugal; 3 Centro Hospitalar Universitário de São João, Alameda Prof. Hernâni Monteiro, 4200-319 Porto, Portugal; 4 Centro de Estatística e Aplicações da Universidade de Lisboa, Faculdade de Ciências, Universidade de Lisboa, Campo Grande, 1749-016 Lisboa, Portugal; 5 Z-Stat4life, Espaço Cowork Baldaya, Palácio Baldaya, Estrada de Benfica N° 701ª, 1549-011 Lisboa, Portugal; 6 Hospital de Braga, Sete Fontes—São Victor, 4710-243 Braga, Portugal; 7 Centro Hospitalar de Trás-os-Montes e Alto Douro, Avenida da Noruega, 5000-508 Vila Real, Portugal; 8 Hospital da Senhora da Oliveira Guimarães, Rua dos Cutileiros, Creixomil, 4835-044 Guimarães, Portugal; 9 Centro Hospitalar Universitário de Santo António, Rua Prof. Vicente José de Carvalho N° 37, 4050-366 Porto, Portugal; 10 Centro Hospitalar do Baixo Vouga, Av. Artur Ravara, 3810-501 Aveiro, Portugal; 11 Unidade Local de Saúde de Castelo Branco, Avenida Pedro Álvares Cabral, 6000-085 Castelo Branco, Portugal; 12 Hospital Professor Doutor Fernando Fonseca, IC 19, 2720-276 Amadora, Portugal; 13 Centro Hospitalar Universitário Cova da Beira, Alameda Pêro da Covilhã, 6200-251 Covilhã, Portugal; 14 Centro Hospitalar de Leiria, Rua das Olhalvas, 2410-197 Leiria, Portugal; 15 Centro Hospitalar Tondela-Viseu, Avenida Rei D. Duarte, 3504-509 Viseu, Portugal; 16 Centro Hospitalar Universitário de Lisboa Central, Rua José António Serrano, 1150-199 Lisboa, Portugal; 17 Centro Hospitalar Universitário Lisboa Norte, Avenida Professor Egas Moniz, 1649-035 Lisboa, Portugal; 18 Instituto Português de Oncologia de Lisboa Francisco Gentil, Rua Professor Lima Basto, 1099-023 Lisboa, Portugal; 19 Centro Hospitalar Universitário de Coimbra, Praceta Professor Mota Pinto, 3004-561 Coimbra, Portugal; 20 Centro Hospitalar de Lisboa Ocidental, Rua da Junqueira N° 126, 1349-019 Lisboa, Portugal; joanapfvasconcelos@hotmail.com; 21 Hospital Distrital de Santarém, Avenida Bernardo Santareno, 2005-177 Santarém, Portugal; 22 Unidade Local de Saúde da Guarda, Avenida Rainha Dona Amélia, 6300-858 Guarda, Portugal; 23 Unidade Local de Saúde do Norte Alentejano, Avenida de Santo António, Apartado 234, 7301-853 Portalegre, Portugal; 24 Centro Hospitalar do Médio Tejo, Avenida Maria de Lourdes de Mello Castro, Ap. 118, 2304-909 Tomar, Portugal; 25 Centro Hospitalar de Vila Nova de Gaia/Espinho, Rua Conceição Fernandes, 4434-502 Vila Nova de Gaia, Portugal; 26 Faculdade de Medicina, Universidade de Lisboa, Avenida Professor Egas Moniz, 1649-028 Lisboa, Portugal; 27 Centro Hospitalar de Setúbal, Rua Camilo Castelo Branco, Apartado 140, 2910-446 Setúbal, Portugal; 28 Hospital de Cascais Dr. José de Almeida, Avenida Brigadeiro Victor Novais Gonçalves, 2755-009 Alcabideche, Portugal; 29 Centro Hospitalar Barreiro Montijo, Avenida Movimento das Forças Armadas, 2834-003 Barreiro, Portugal; 30 Centro Hospitalar do Tâmega e Sousa, Avenida do Hospital Padre Américo, N° 210, Guilhufe, 4560-136 Penafiel, Portugal; ferrazrit@gmail.com; 31 Instituto Português de Oncologia de Coimbra Francisco Gentil, Avenida Bissaya Barreto N° 98, 3000-075 Coimbra, Portugal; 32 Hospital Beatriz Ângelo, Avenida Carlos Teixeira, N° 3, 2674-514 Loures, Portugal; 33 Hospital de Vila Franca de Xira, Estrada Carlos Lima Costa N°2, 2600-009 Vila Franca de Xira, Portugal; 34 Unidade Local de Saúde do Nordeste, Avenida Abade de Baçal, 5301-852 Bragança, Portugal; 35 Centro Hospitalar Universitário do Algarve, Rua Leão Penedo, 8000-386 Faro, Portugal; 36 Hospital Garcia de Orta, Avenida Torrado da Silva, 2805-267 Almada, Portugal

**Keywords:** *Leishmania*, leishmaniasis, cutaneous, mucosal, Portugal, 2010–2020

## Abstract

*Leishmania infantum*, a zoonotic vector-born parasite, is endemic in the Mediterranean region, presenting mostly as visceral (VL), but also as cutaneous (CL) and mucosal leishmaniasis (ML). This study aimed to describe the epidemiological and clinical aspects of the CL and ML cases diagnosed in mainland Portugal between 2010 and 2020. Collaboration was requested from every hospital of the Portuguese National Health System. Cases were screened through a search of diagnostic discharge codes or positive laboratory results for *Leishmania* infection. Simultaneously, a comprehensive literature search was performed. Descriptive statistics and hypothesis testing were performed using IBM^®^ SPSS^®^ Statistics. A total of 43 CL and 7 ML cases were identified, with a predominance of autochthonous cases (86%). In CL, immunosuppressed individuals constituted a significant proportion of patients (48%), and in this group, disseminated CL (22%) and simultaneous VL (54%) were common. In autochthonous cases, lesions, mostly papules/nodules (62%), were frequently observed on the head (48%). The approach to treatment was very heterogeneous. ML cases were all autochthonous, were diagnosed primarily in older immunosuppressed individuals, and were generally treated with liposomal amphotericin B. The findings suggest a need for enhanced surveillance and reporting, clinical awareness, and diagnostic capacity of these forms of leishmaniasis to mitigate underdiagnosis and improve patient outcomes. A holistic One Health approach is advocated to address the multifaceted challenges posed by leishmaniases in Portugal and beyond.

## 1. Introduction

Leishmaniases are a group of diseases caused by protozoan parasites of the genus *Leishmania*. These parasites are transmitted by infected female phlebotomine sand flies, and the disease is zoonotic in most settings [1]. The clinical spectrum of symptomatic disease is usually grouped into two main syndromes, visceral leishmaniasis (VL) and cutaneous leishmaniasis (CL) [1], both of which are endemic and geographically widespread in the Mediterranean region. In this region, *L. infantum*, which belongs to the *L. donovani* complex, is the etiologic species of most autochthonous human leishmaniasis cases [2]. Infection with *L. infantum*, when symptomatic, usually presents as VL, although cases of simultaneous or independent CL and mucosal leishmaniasis (ML) caused by this species are increasingly recognized [3]. In the western Mediterranean regions where *L. infantum* is endemic, including in Portugal, *Phlebotomus perniciosus* is the main vector [4], and dogs are the main reservoir for human infection [5].

Cutaneous leishmaniasis is traditionally considered a rare disease in Portugal, with very few cases documented prior to 2002 [6]. However, since reporting of CL is not mandatory, information regarding cases of this clinical form is dispersed between databases of different hospitals and a few publications in national and international journals and master/doctoral theses. Most cases described after 2000 are locally acquired and likely caused by *L. infantum* (even though species identification was seldom reported [7,8]); few cases suggested imported disease by dermotropic species from the New World (including those from the *L. Viannia* subgenus [9]). ML cases caused by endemic *L. infantum* have also been described [10,11,12]. In contrast to other European endemic countries, such as France, where CL cases are mostly imported [13], in Portugal, most published cases seem to be autochthonous. In most of these countries, however, CL is not regularly monitored at a national level [2]; in Portugal, no reports or reviews addressed CL nationally, and only one did so at a regional level (in Cova da Beira and Beira Interior Norte, describing 13 patients [14]). Consequently, the epidemiology of CL in Portugal is largely unknown, and attention should be focused on understanding the contexts of endemic disease, as well as the trends in imported cases following recent waves of migration from and increased travel to dermotropic species endemic countries, especially those in South America [15].

Therefore, this study aimed to describe the cases of cutaneous or mucosal leishmaniases diagnosed in hospitals of the Portuguese National Health Service between 2010 and 2020, or reported in the scientific literature in an equivalent period.

## 2. Materials and Methods

### 2.1. Study Population

Mainland Portugal is located in southwest Europe, bordering Spain and the Atlantic Ocean, and is divided into seven NUTS2 regions and 24 NUTS3 regions [16]. According to the 2021 national census, the population of mainland Portugal was 9,857,593 inhabitants [17]. Between 2010 and 2020, hospital-based healthcare services were provided by the Portuguese National Health Service (NHS) in approximately 100 general and specialized hospitals in mainland Portugal, according to data from the Directorate-General for Health (DGS) of Portugal [18]. Some of these hospitals are grouped into hospital centers. By protocol, every episode of emergency or hospitalization in these hospitals is given a code on discharge for primary and secondary diagnoses, following the International Classification of Diseases (ICD).

In this multicenter retrospective study, individuals diagnosed with cutaneous and/or mucosal leishmaniases in one of the hospitals of the Portuguese NHS, located in mainland Portugal, between 2010 and 2020, inclusively, were included in this study. Only laboratory confirmed cases were included, which consisted of the presence of a compatible clinical picture and meeting at least one of the following criteria: (i) detection of *Leishmania* DNA in cutaneous and/or mucosal samples; (ii) visualization of intracellular organisms in macrophages, compatible with *Leishmania* amastigotes in biopsy material or cytological examination; (iii) growth of *Leishmania* from a clinical sample inoculated in a specific culture medium.

### 2.2. Data Collection

Every hospital or hospital center of the NHS in mainland Portugal was contacted, and collaboration in this study was requested. Cases in each included hospital were screened through a search of the following diagnostic discharge codes: 085, 085.1, 085.2, 085.3, 085.4, 085.5, 085.9 (ICD-9); B55, B55.1, B55.2, B55.9 (ICD-10). In hospitals where codification of diagnosis was incomplete or unavailable for the whole or parts of the period of analysis, the listing of cases was complemented by searching skin/mucosa samples in which *Leishmania* DNA was detected by PCR and cytology, as well as histopathology reports in which observation of *Leishmania* amastigotes was mentioned. Sociodemographic and clinical data for the cases identified was extracted from the medical records of each episode, codified, and inserted into a digital database. Data extraction was carried out by different professionals; a common database was used, and a protocol for filling in the required information was provided to every collaborator.

Additionally, a comprehensive literature search was performed on 3 March 2024 by sourcing National Library of Medicine (NLM) resources through PubMed (https://pubmed.ncbi.nlm.nih.gov/, accessed on 3 March 2024) using the following Boolean string: (“cutaneous” OR “mucosal” OR “mucocutaneous”) AND “leishmaniasis” AND “Portugal”. Search results were saved as a comma-separated value (CSV) file, and subsequently imported into Microsoft Excel^®^ (Version Office 365, Microsoft Corp, Redmond, USA) Study eligibility was manually assessed. All records were screened according to the title and abstract, if available. Only studies published between 2011 and 2022 and in which at least one of the affiliations of the authors was a Portuguese hospital were included. This time frame was selected to match the cases diagnosed in the hospitals between 2010 and 2020, considering a 1–2 year delay between the diagnosis and publishing of the cases. Only case reports or series of confirmed cutaneous and/or mucosal leishmaniases were retained, including those published in English or Portuguese languages.

Cases of CL/ML obtained from the two sources (hospitals and publications) were matched, considering the following individual details, whenever available: age and sex of patient, comorbidities, region of residence at the time of diagnosis, year of admission to the hospital, diagnostic techniques, and treatment strategy. For duplicated cases, data from both sources were merged into a single entry in the final database. Categorical variables extracted from the clinical records or scientific articles were analyzed, mostly using the categories provided as options in the standardized database, but regrouping was performed in some cases. NUTS regions and municipalities were defined according to the latest organizational definition, implemented in 2024. The term “migrant” was used for people born abroad. Cases were defined as autochthonous if no species other than *L. infantum* was identified and if (a) there was no lifetime history of travel or residence abroad in CL endemic regions (any of the countries listed as endemic by the WHO for 2022 [19]); (b) there was a history of travel or residence in CL endemic region(s), but it occurred more than 12 months before the beginning of symptoms, and there was no change in immune status since the stay abroad; (c) or there was no information regarding travel history. Cases not meeting any of these criteria were considered as imported. Time to presentation represented the amount of time elapsed since the beginning of signs/symptoms related to leishmaniasis and the first visit to healthcare providers/institutions. Time to diagnosis represented the amount of time elapsed since presentation to healthcare providers and the confirmation of the diagnosis of leishmaniasis (according to the criteria above). Time to treatment was measured as the amount of time elapsed since the confirmation of diagnosis and the start of *Leishmania* directed therapy. For the purposes of this study, a patient was considered immunosuppressed if one or more of the following conditions were present: HIV infection with a CD4 cell count <500/µL; any primary immunodeficiency; active solid or hematologic malignancy; prior solid organ or bone marrow transplantation; current treatment with immunosuppressive/immunomodulatory drugs (as listed in [20]). The types of lesions were defined according to clinical records. Disseminated CL was defined by the presence of over 10 lesions in multiple non-contiguous sites; MCL (mostly associated with *L. braziliensis* complex) was defined as a condition in which, following (or simultaneously with) a non-adjacent primary cutaneous lesion, parasites disseminate towards the mucosa; ML was defined as a condition in which localized *Leishmania* lesions in the mucosa occur without primary skin involvement, or in which skin involvement presents concurrently only in contiguous areas [21]. Non-improvement was defined as persistence or worsening of signs/symptoms, despite appropriate therapy, and was assessed at seven and thirty days after starting treatment. These two timeframes were defined by the authors to allow homogeneous data collection regarding outcome in the different hospitals involved. European guidelines propose a definition of non-response for CL as no clinical improvement at four weeks after start of therapy [21]. Relapses were defined as recurrence of signs/symptoms and positive culture/PCR/microscopy in a skin/mucosa sample after completing primary treatment with clinical improvement at 30 days.

### 2.3. Statistical Analysis

Mean annual incidence of CL was estimated based on the following formula: Incidence = (New Cases)/(Population × Timeframe), considering a timeframe of 11 years and an at-risk population, for each region, consisting of the average value between the number of inhabitants estimated in the census of 2011 and the census of 2021, according to the National Institute of Statistics [17]. The corresponding 95% confidence intervals (CIs) for the incidence rate were obtained using a substitution method [22].

Descriptive statistics and hypothesis testing were performed using IBM^®^ SPSS^®^ Statistics Version 29.0. Bar charts were built using Microsoft^®^ Excel^®^. Geographical representation and analysis of results were achieved using QGIS^®^ Version 3.22.

For categorical variables, absolute frequencies and percentages were calculated. Symmetric continuous variables were summarized by means with standard deviations, and asymmetric continuous variables (e.g., age, lesion size) by medians with interquartile intervals (IQIs). Missing or unknown data were excluded from denominators, unless stated otherwise.

Comparisons between CL and ML were performed using the Pearson Chi-square test (CST) for categorical variables, or Fisher’s exact test (FET), in case of failure of the assumptions of the CST. For continuous variables, after checking the assumptions of normality and homogeneity of the variances, the Mann–Whitney U test (MWT) was used for comparing two independent groups.

## 3. Results

Data from 42 of the 45 hospitals or hospital centers in mainland Portugal were available for analysis. A total of 42 cases of CL and 7 cases of ML were diagnosed between 2010 and 2020 in the hospitals included. A total of 79 articles were obtained from the PubMed database search. Of these, six articles were selected, according to selection criteria, representing a total of three cases of CL [8,9,23] and three cases of ML [10,11,12]. Of these cases, five were matched with cases retrieved through the hospital searches. Consequently, combining the two sources of data, 43 cases of CL and 7 cases of ML were available for analysis.

### 3.1. Sociodemographic Characteristics and Comorbidities

Sociodemographic characteristics of CL/ML cases are represented in Table 1. The median age was 48 years old (IQI 33–61.2) and was significantly higher in ML patients compared to CL patients (66 vs. 47, *p* = 0.026, U = 71.0). Male sex was predominant globally and in both forms of leishmaniasis. Seven cases of CL (16.3%) were imported (from Brazil *n* = 3, Morocco *n* = 2, Mexico *n* = 1, Tunisia *n* = 1); all cases of ML were autochthonous. Migrants represented approximately 25% of the patients diagnosed.

Immunosuppressing conditions were present in 48.0% of patients, including HIV infection/AIDS, reported in nearly one-third of patients (78.6% had CD4 cell counts <200/µL). Additionally, chronic pharmacologic immunosuppression for inflammatory diseases was reported in 12.2% of patients (*n* = 6), most commonly consisting of regimens containing anti-TNFα (*n* = 2) and methotrexate (*n* = 3). Chronic organ dysfunction was present in 28.6% of patients, especially those with ML (*p* = 0.005, FET).

**Figure 1:**
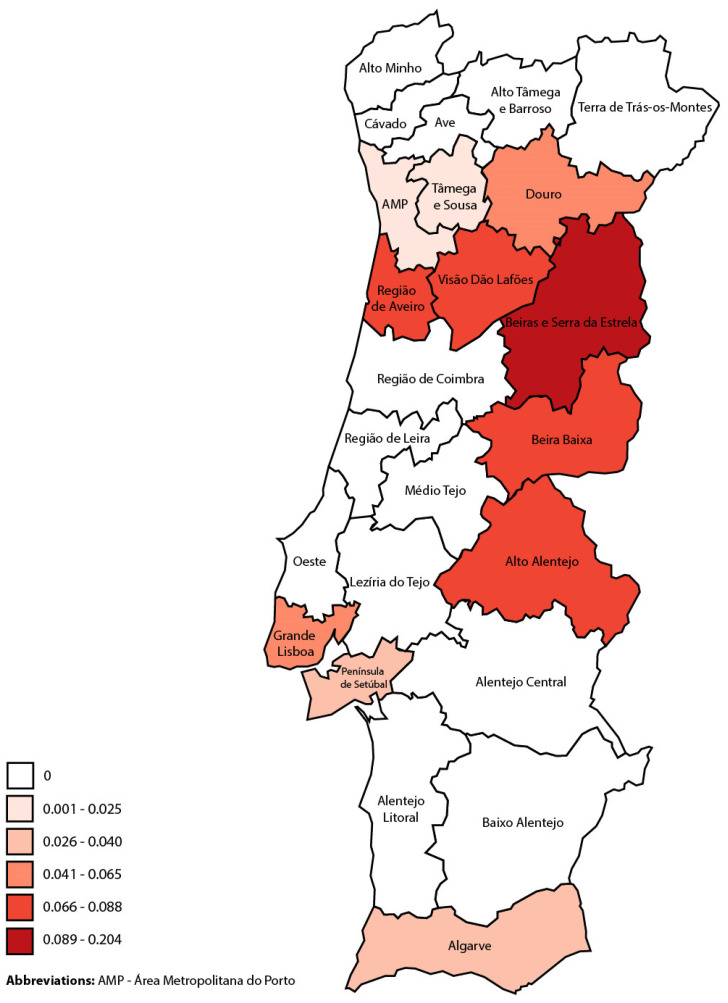
Estimated mean annual incidence, per 100,000 population, between 2010 and 2020 of cutaneous leishmaniasis by NUTS3 region.

Of the 50 cases of CL/ML identified, 46 were considered primary (or incident) cases, and 4 were relapsing cases (first episode diagnosed before 2010). The estimated annual incidence of CL/ML in mainland Portugal between 2010 and 2020 was 0.036 cases/100,000 population/year. Table 2 and Figure 1 show the number of cases of CL/ML diagnosed between 2010 and 2020 and the estimated annual incidence in this period by NUTS2 and NUTS3 region.

### 3.2. Clinical Aspects of CL

Globally, the median time from onset of symptoms to presentation to healthcare services was 16 weeks (IQI 8–48), and the median time from presentation to diagnosis was 16 days (IQI 7–35). Hospital admission occurred in 60.5% of patients and was significantly more common in immunosuppressed patients (77.3% of cases; *p* = 0.021, χ^2^ = 5.324, df = 1).

The clinical aspects of autochthonous cases of CL (*n* = 36) are represented in Table 3. Approximately 40% of patients had multiple lesions; the median size of the largest lesion was 30 mm (IQI 10–40). The most common type of lesion was a papule/nodule (present in 62.1% of patients), followed by an ulcer (24.1%). The head was the most common anatomical location (48.3%), followed by the upper and lower limbs (each in 31.0%). Skin bacterial superinfection was reported in 11.1% of cases. The lesions were similar in immunosuppressed and non-immunosuppressed patients, except that they were more frequently localized in the trunk (*p* = 0.002, FET) and were painful (*p* = 0.037, FET) in the former group. Additionally, 54.5% of immunosuppressed patients had simultaneous VL, contrasting with 4.2% in the non-immunosuppressed group (*p* < 0.001, CST, χ^2^ = 14.369, df = 1). Disseminated CL was only seen in immunosuppressed patients, occurring in 22.2% of these cases (*n* = 4, all of them people living with HIV).

**Figure 2:**
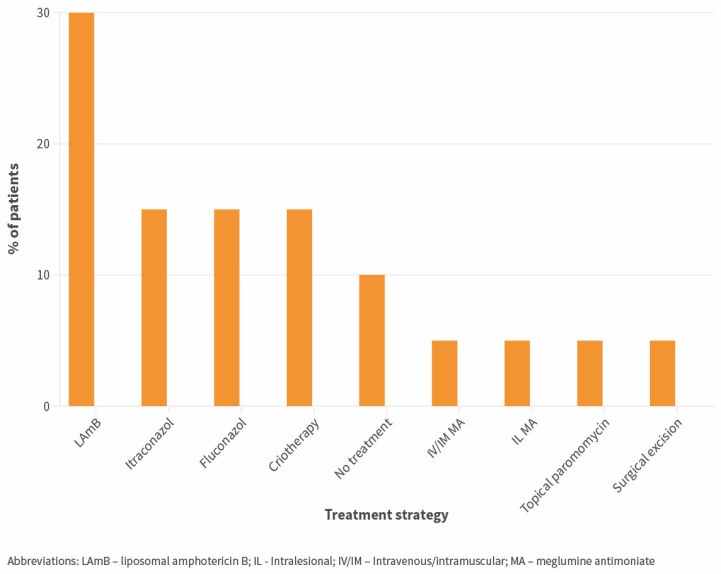
Strategies used for primary treatment of patients with autochthonous exclusively cutaneous leishmaniasis (no evidence of simultaneous visceral involvement) (*n* = 20).

Skin samples of autochthonous CL cases were mostly obtained by biopsy, and by smear/scrapping in only in 2.4% of cases. Microscopy was used in all cases and was positive in 100% of these cases. PCR was used in 28.6% of cases and was positive in 85.7% of these. *Leishmania* species/complex was identified in 19.4% of cases (by molecular biology techniques); all belonged to the *L. donovani* complex (*n* = 6). Successful identification was available for 4/7 imported cases (*L. donovani* complex: *n* = 2, *L. Viannia* sp.: *n* = 2). Serology was used as part of the diagnostic workup in 34.6% of cases and was positive in 37.5% of these. Treatment for CL was introduced in 93.8% of patients, and the median time from diagnosis to treatment was four days (IQI 0–31). Most patients were treated with monotherapy (92.5%). Systemic treatment was used in 80.0% of patients and significantly more frequently in immunosuppressed patients (*p* = 0.033, FET). Detailed strategies used for primary treatment of patients who had exclusively CL (without simultaneous visceral involvement) (*n* = 20) are represented in Figure 2. Improvement by day 7 or 30 after starting therapy was mentioned in 60.0 and 76.2% of patients, respectively, and was not significantly different in immunosuppressed patients. Switching to a different regimen or retreatment due to non-improvement was implemented in 19.2% of patients. Relapse was reported in two patients, both immunosuppressed.

Time from presentation to diagnosis was the only variable that differed significantly between NUT2 regions (*p* = 0.037, KWT = 6.616, df = 2), being shorter in the Norte, followed by AML and Centro.

### 3.3. Clinical Aspects of ML

Leishmaniasis with mucosal involvement (*n* = 7) represented ML in all cases (no cases of MCL were identified). One patient was a person living with HIV, three were immunosuppressed for other reason, and two were non-immunosuppressed adults (one not defined). In five cases, only the nasal mucosa was involved (one with septal perforation), and in one case, both the oral and the nasal mucosa were involved. All cases were diagnosed by biopsy of the lesions and identification of amastigotes using microscopy, and PCR was used additionally in five cases. All cases were treated with liposomal amphotericin B except one, which was treated with IV meglumine antimoniate. One patient, who presented with simultaneously VL, died.

## 4. Discussion

The present study reinforces that the incidence of autochthonous CL cases seems to be lower than that of VL at a national level [24], although marked differences were noted between the NUTS2 and NUTS3 regions. Few data were previously available in the country and were mostly derived from case reports and case series, since no national reporting system is in place [25]. These data already suggested that the Beiras e Serra da Estrela region could be an important focus of CL [14], which was also pointed out in the present study. Recent cases revealed in this study in regions where CL has not been previously described in the literature, such as the Aveiro region, should be further investigated. Additionally, further studies could help elucidate whether or not this heterogeneity could be explained by an increased clinical awareness in certain regions or the presence of a distinct, particularly dermotropic *L. infantum* genotype. In support of the first hypothesis, it should be noted that in areas of neighboring Spain, the incidence of autochthonous CL was similar to that of VL [26]; however, CL notification is mandatory in Spain, as opposed to the protocol for Portugal [2] and in the present study, the underestimation of CL incidence could be due to inadequate coding at the hospital level and insufficient laboratory information.

Imported CL still represents a minority of cases in Portugal, as opposed to other European endemic countries, such as (metropolitan) France [27]; however, this could be expected to change in upcoming years due to increasing migration from CL endemic countries, such as Brazil [15]. Since no systematic clinical screening program is implemented in migrant populations in Portugal, leishmaniasis cases, especially (spontaneously resolving) CL, could go unnoticed and translate into an underestimation of imported cases.

In the autochthonous CL group, 48.0% were immunosuppressed, and in this group, 54.5% presented with simultaneously VL, which suggests that immunosuppressed people with CL benefit from a more intensive diagnostic workup in a setting where most CL is assumed to be caused by *L. infantum* [28]. Overall, time from onset of symptoms to presentation to healthcare and time from presentation to diagnosis were long, likely reflecting, on one hand, low concern of the patients for the lesions and, on the other hand, the low awareness of clinicians of the disease, or their unfamiliarity with the availability and performance of diagnostic techniques. In terms of lesion characteristics, the findings of the present study overlap those of other case series in the Mediterranean context, where *L. infantum* is endemic and includes frequent multiple lesions, a lower prevalence of ulcerated lesions compared to nodules/papules or plaques, and a predominance of head/neck lesions [3,29].

According to the present study, the diagnosis of CL in Portugal between 2010 and 2020 relied mostly on microscopy. As PCR was performed in only 42.1% of cases, *Leishmania* species was not identified in all imported cases, and therapy was commonly selected based on the probable geographical location of the infection. However, species identification in New World CL could have implications for individual management, especially in areas where multiple species co-circulate, since species in the *Viannia* subgenus, especially *L. braziliensis*, have been more frequently associated with MCL, requiring initial screening for this form and longer follow-up time [30]. On the other hand, species identification in Old World CL could be of public health interest, in the context of surveillance and assessment of risk of introduction of species such as dermotropic *L. tropica*. This anthroponotic species has been reported as the most imported species from refugees [31], and Phlebotomus sergenti, a specific vector of *L. tropica*, is widely distributed in Southern Europe, including in Portugal [32].

Treatment of CL cases was very heterogeneous but often relied on systemic therapy, especially IV/IM in immunosuppressed patients, which is in accordance with European recommendations [21]. Overall, rates of non-improvement (or failure) at 30 days after starting treatment (23.8%) were similar to those reported in previous studies in settings where *L. infantum* is endemic [3].

Although mucosal involvement is more commonly associated with *L.* (*Viannia*) sp., ML is increasingly recognized in the Old World in the context of *L. infantum* infection and especially in immunosuppression [33]. In the present study, seven cases were identified, all were autochthonous, and two were not immunosuppressed. Nose and throat physicians in endemic regions should be alert to the presentation, diagnostic approach, and management specificities of ML, particularly in regards to non-immunosuppressed patients and lesions located in the oral, pharyngeal, and laryngeal mucosa.

Finally, this study presents some limitations, beginning with the fact that in some hospitals, not all information was collected due to a lack of collaboration or the absence of patient consent. The coding of the diagnosis for inpatients was not uniformly performed and digitalized in every hospital for the whole duration of the study period, and coding for outpatients was irregularly performed in hospitals, so cases were screened via laboratory results, whenever feasible. Some hospitals required internal personnel to access information, so in some cases, interpretation of variables could be different, despite using the same database. Regarding the literature review of CL cases diagnosed in Portugal, a notable limitation may arise from the possibility that further cases might have been reported solely in national journals not indexed in PubMed or exclusively presented at conferences or congresses.

## 5. Conclusions

This study sheds light on the epidemiological and clinical landscape of CL and ML in Portugal between 2010 and 2020. While the incidence of autochthonous CL was low, CL was more common than previously reported, but still less common than in neighboring countries, possibly translating into significant underdiagnosis. Regional disparities highlight the importance of localized surveillance efforts. Programs to control leishmaniasis should focus not only on reducing underreporting, but also on raising awareness for the disease’s different clinical forms among healthcare practitioners and providing tools for earlier diagnosis. Clinical suspicion should be particularly heightened for immunosuppressed people, who are disproportionately affected.

The present findings also underscore the potential for the underestimation of imported CL cases, particularly in the context of increasing migration from endemic regions, in the absence of systematic clinical screening programs. Given the implications for individual management and public health surveillance, there is a need for greater emphasis on species identification in imported cases.

Systematically combining clinical and national surveillance data could allow for a more detailed assessment of the epidemiologic situation and an evaluation of the progress in clinical practice, uncovering gaps that need to be addressed in the near future. In order to improve the overall outcome for leishmaniasis patients, human data should also be integrated with data from vectors and mammal hosts to produce holistic strategies to control the disease in several stages of the life cycle, following a One Health approach.

## Figures and Tables

**Table 1:** Sociodemographic characteristics and comorbidities of leishmaniasis cases, globally and by form of disease.

	Global	CL	ML	*p*-Value
Number	50	43	7	
Median age, years (IQI)	48	47	66	0.026 *
	[33–61.25]	[33–59]	[50–75]	(U = 71.0)
Male sex (%)	68.0	69.8	57.1	0.666
	(34/50)	(30/43)	(4/7)	(FET)
Country of birth (%)				
Native	75.6	72.5	100	0.313 (FET)
	(34/45)	(29/40)	(5/5)	
Migrant	24.4	27.5	0	
	(11/45)	(11/40) ^a^	(0/5)	
Origin of infection (%)				
Autochthonous	86.0	83.7	100	0.573 (FET)
	(43/50)	(36/43)	(7/7)	
Imported	14.0	16.3	0	
	(7/50)	(7/43) ^b^	(0/7)	
Immunosuppression (%)				
Yes	48.0	46.5	57.1	0.697
	(24/50)	(20/43)	(4/7)	(FET)
Unknown/Not reported	2.0	0	14.3	
	(1/50)	(0/43)	(1/7)	
HIV infection/AIDS				
Yes (%)	32.7	34.9	16.7	0.649
	(16/49)	(15/43)	(1/6)	(FET)
CD4 cell count <200/µL (%)	78.6	84.6	0	0.214
	(11/14)	(11/13)	(0/1)	(FET)
Chronic pharmacologic immunosuppression (%)				
Inflammatory/autoimmune diseases ^c^	12.2	9.3	33.3	0.151
	(6/49)	(4/43) ^d^	(2/6) ^e^	(FET)
Other	4.1	2.3	16.7	
	(2/49)	(1/43) ^f^	(1/6) ^g^	
Chronic dysfunction/condition (%)				
Yes	28.6	19.4	83.3	0.005 *
	(12/42)	(7/36) ^h^	(5/6) ^i^	(FET)
^a^ Brazil: *n* = 4; Cape Verde: *n* = 2; Guinea-Bissau: *n* = 1; Morocco: *n* = 1; São Tomé e Príncipe: *n* = 1; Senegal: *n* = 1; unknown: *n* = 1				
^b^ Brazil: *n* = 3; Morocco: *n* = 2; Mexico: *n* = 1; Tunisia: *n* = 1				
^c^ Systemic lupus erythematosus: *n* = 2; ankylosing spondylitis: *n* = 1; Crohn’s disease: *n* = 1; psoriasis: *n* = 1; rheumatoid arthritis: *n* = 1				
^d^ Adalimumab: *n* = 1; adalimumab + methotrexate: *n* = 1, methotrexate: *n* = 1; methotrexate + prednisolone: *n* = 1				
^e^ Methotrexate: *n* = 1; mycophenolate mofetil + prednisolone: *n* = 1				
^f^ Lymphoma: *n* = 1				
^g^ Kidney transplant: *n* = 1 ^h^ Chronic kidney disease *n* = 6; chronic heart failure *n* = 1; chronic obstructive respiratory disease *n* = 1; chronic hepatic disease *n* = 1; diabetes mellitus *n* =1				
^i^ Chronic kidney disease: *n* = 2; chronic heart failure: *n* = 1; chronic obstructive respiratory disease: *n* = 1; diabetes mellitus: *n* = 3 * Statistically significant				

Abbreviations: CL—cutaneous leishmaniasis; ML—mucosal leishmaniasis; HIV—human immunodeficiency virus; AIDS—acquired immunodeficiency syndrome; IQI—interquartile interval; FET—Fisher’s exact test.

**Table 2:** Number of cases of cutaneous and mucosal leishmaniasis diagnosed between 2010 and 2020, inclusively, and mean annual incidence in this period, per 100,000 population, by NUTS2 and NUTS3 region.

Region	Average Population in 2011–2021 *	Number of CL/ML Cases	Mean Annual CL/ML Incidence **	95% CI
Mainland Portugal	9,951,765	39	0.036	0.024–0.047
Norte	3,638,134	7	0.017	0.006–0.033
Alto Minho	238,051	0	0	NA
Cávado	413,387	0	0	NA
Ave	421,933	0	0	NA
Área Metropolitana do Porto	1,747,876	4	0.021	0.003–0.046
Alto Tâmega	89,195	0	0	NA
Tâmega e Sousa	420,776	1	0.022	0.001–0.120
Douro	194,516	1	0.047	0.001–0.260
Terras de Trás-os-Montes	112,399	0	0	NA
Centro	1,695,204	13	0.070	0.037–0.119
Região de Aveiro	368,898	3	0.074	0.015–0.216
Região de Coimbra	448,500	0	0	NA
Região de Leiria	290,692	0	0	NA
Viseu Dão Lafões	260,205	2	0.070	0.008–0.252
Beira Baixa	103,597	1	0.088	0.002–0.489
Beiras e Serra da Estrela	223,312	5	0.204	0.066–0.475
Oeste e Vale do Tejo	823,948	0	0	NA
Oeste	363,025	0	0	NA
Médio Tejo	219,266	0	0	NA
Lezíria do Tejo	241,657	0	0	NA
Grande Lisboa	2,052,392	13	0.058	0.031–0.098
Península de Setúbal	793,651	3	0.034	0.007–0.100
Alentejo	489,259	1	0.019	0.000–0.069
Alentejo Litoral	97,183	0	0	NA
Baixo Alentejo	120,777	0	0	NA
Alto Alentejo	111,714	1	0.081	0.002–0.453
Alentejo Central	159,585	0	0	NA
Algarve	459,174	2	0.040	0.005–0.143

* Arithmetic mean between the population size estimated in the National Census of 2011 and 2021. ** Number of new cases per 100,000 population, per year, based on the following formula: Incidence = (New Cases)/(Population × Timeframe). Abbreviations: CL—cutaneous leishmaniasis; ML—mucosal leishmaniasis; NA—not applicable; CI—confidence interval.

**Table 3:** Clinical presentation and management of autochthonous cutaneous leishmaniasis cases between 2010 and 2020.

Type of Lesion (%)	
Papule/Nodule	62.1
	(18/29)
Ulcer	24.1
	(7/29)
Macule/Plaque	20.7
	(6/29)
Multiple lesions (%)	41.9
	(13/31)
Median size of largest lesion, mm (IQI)	30
	[10–40]
Location of lesions (%)	
Head	48.3
	(14/29)
Upper limbs	31.0
	(9/29)
Lower limbs	31.0
	(9/29)
Trunk	20.7
	(6/29)
Disseminated cutaneous leishmaniasis (%)	12.5
	(4/32)
Local pain (%)	25.0
	(5/20)
Skin superinfection ^a^ (%)	11.1
	(3/27)
Simultaneous visceral leishmaniasis (%)	38.9
	(14/36)
Technique used in skin/mucosa sample (%)	
Microscopy	100
	(31/31)
Positive result	100
	(30/30)
Polymerase chain reaction	28.6
	(8/28)
Positive result	85.7
	(6/7)
Identification of species (%)	19.4
	(6/31)
Serology (%)	
Yes ^b^	34.6
	(9/26)
% positive	37.5
	(3/8)
Treatment of primary episode (%)	
Yes	93.8
	(30/32)
Median time from diagnosis to treatment, days (IQI)	4
	[0–31]
Monotherapy	93.3
	(28/30)
Systemic	80.0
	(24/30)
Topical	23.3
	(7/30)
Side effects	15.4
	(2/13)
Outcome of treatment (%)	
Improvement at 7 days	60.0
	(9/15)
Improvement at 30 days	76.2
	(16/21)
Switch of treatment/retreatment (non-improvement)	19.2
	(5/26)
Relapse	5.6
	(2/36)
^a^ methicillin-sensitive *Staphylococcus aureus: n* = 1; Pseudomonas aeruginosa: *n* = 1; non-identified *n* = 1	
^b^ immunofluorescent antibody test: *n* = 5; unknown: *n* = 4	

Abbreviations: IQI—interquartile interval.

## Data Availability

The datasets generated and analyzed during the current study are not publicly available due to a confidentiality commitment with the health institutions and the participants.
